# Aging effects on the encoding/retrieval flip in associative memory: fMRI evidence from incidental contingency learning

**DOI:** 10.3389/fnagi.2024.1357695

**Published:** 2024-03-13

**Authors:** Else Schneider, Marko Rajkovic, Rudolf Krug, Marco P. Caviezel, Carolin F. Reichert, Oliver Bieri, André Schmidt, Stefan Borgwardt, Thomas Leyhe, Christoph Linnemann, Annette B. Brühl, Undine E. Lang, Tobias Melcher

**Affiliations:** ^1^Center of Affective, Stress and Sleep Disorders (ZASS), University Psychiatric Clinics (UPK), University of Basel, Basel, Switzerland; ^2^Center of Old Age Psychiatry, University Psychiatric Clinics (UPK), University of Basel, Basel, Switzerland; ^3^Transfaculty Research Platform Molecular and Cognitive Neuroscience, University of Basel, Basel, Switzerland; ^4^University Psychiatric Clinics (UPK), University of Basel, Basel, Switzerland; ^5^Centre for Chronobiology, University Psychiatric Clinics (UPK), University of Basel, Basel, Switzerland; ^6^Division of Radiological Physics, Department of Radiology and Nuclear Medicine Clinic, University Hospital Basel, University of Basel, Basel, Switzerland; ^7^Department of Clinical Research (DKF), University Psychiatric Clinics (UPK), Translational Neurosciences, University of Basel, Basel, Switzerland; ^8^Translational Psychiatry Unit (TPU), Department of Psychiatry and Psychotherapy, University of Lübeck, Lübeck, Germany

**Keywords:** associative memory, incidental learning, age-effect, encoding/retrieval flip, anterior insular cortex, posterior midline region, middle temporal gyrus

## Abstract

**Introduction:**

Associative memory is arguably the most basic memory function and therein constitutes the foundation of all episodic and semantic memory processes. At the same time, the decline of associative memory represents a core feature of age-related cognitive decline in both, healthy and pathological (i.e., dementia-related) aging. The neural mechanisms underlying age-related impairments in associative memory are still not fully understood, especially regarding incidental (i.e., non-intentional) learning.

**Methods:**

We investigated the impact of age on the incidental learning and memory retrieval of face-name combinations in a total sample of 46 young (*N* = 23; mean age = 23.39 years) and elderly (*N* = 22, mean age = 69.05 years) participants. More specifically, particular interest was placed in age-related changes in encoding/retrieval (E/R) flips, which denote a neural antagonism of opposed activation patterns in the same brain region during memory encoding and retrieval, which were assessed using fMRI.

**Results:**

According to our hypothesis, the results showed a significant age-related decline in the retrieval performance in the old group. Additionally, at the neural level, we discovered an abolished E/R flip in the right anterior insula and a joint but reduced E/R flip activation magnitude in the posterior middle cingulate cortex in older subjects.

**Discussion:**

In conclusion, the present findings suggest that the impaired neural modulation of the E/R flip in the right aIC might be a sensitive marker in the early detection of neural aging.

## 1 Introduction

Human knowledge and memory are traditionally conceptualized as cognitive information networks, consisting of nodes and edges of different degrees and weights, respectively (Raaijmakers and Shiffrin, 1980; Eichenbaum, 2004; Zhang et al., 2016). The associative memory (AM) can be considered the basic “control module” of these *“cognitive information networks,”* which enables structural modifications (e.g., adding new information or modulating association strengths) as well as the retrieval of included information. In this function, the AM constitutes the foundation of all episodic and semantic memory processes and represents an integrated part of information processing and related mental operations like, e.g., inferential reasoning (Eichenbaum, 2004). At the same time, the loss of AM represents a core aspect of age-related cognitive decline in both, normal and pathological (i.e., dementia-related) aging (Naveh-Benjamin and Mayr, 2018). In this context, a conclusive body of empirical studies demonstrates that older people may have severe difficulties to form and retrieve associations between items, while their capacity to remember the corresponding single information units may be widely preserved (Old and Naveh-Benjamin, 2008). This specificity of AM reductions in the course of aging is conceptually elaborated in the Associative Deficit Hypothesis (ADH) by Naveh-Benjamin (2000).

Concerning the functional neuroanatomy, both, AM encoding and retrieval, inevitably rely on the medial temporal lobe, i.e., the hippocampus and its adjacent cortical regions (Alvarez and Squire, 1994; Moscovitch et al., 2005). Recent research, however, expands the focus of attention to the functional contribution of other neo-cortical areas to AM, particularly the posterior midline region (PMR), which comprises the middle and posterior middle cingulate cortex (pMCC) and the adjacent precuneus. The PMR shares dense reciprocal connections with the medial temporal lobe (Beckmann et al., 2009; Natu et al., 2019) and can therefore be considered an integral part of the neural memory system. In prior fMRI studies, the PMR consistently showed opposed patterns of neural activation in relation to mnemonic encoding and retrieval processes, mostly with deactivation during encoding and activation during recall or retrieval (Daselaar et al., 2009; Vannini et al., 2011). This prominent finding has been coined as “encoding/retrieval (E/R) flip” and has been replicated in many studies under different settings (for review, see Huijbers et al., 2012). Recent research suggests that the age-specific decline of AM capacity is functionally related to aberrations in the E/R flip (*cf.* Fu et al., 2020). More specifically, the ability to modulate PMR activation in a context-sensitive manner has been shown to decline with increasing age and thereby to be related to significant decrements in memory performance (Vannini et al., 2013; Amlien et al., 2018). However, the neural mechanisms underlying age-related modifications in AM are still not sufficiently understood, and further research is needed to improve our understanding of the impact of age on the neural underpinning of memory.

In a recent study, we examined the neural mechanisms of incidental AM, i.e., the memory for non-intentionally learned associative information, and found an E/R flip not only in the PMR, but also in the right anterior insular cortex (aIC) (Caviezel et al., 2020). Thereby, the anterior insula exhibited the established pattern of deactivation during encoding and activation during retrieval, while the activation pattern in the PMR was inverse compared to prior E/R flip findings. Additional functional connectivity analyses using psycho-physiological interactions (PPIs) revealed a negative coupling between the two E/R flips, in terms that increased aIC activation was related to increased PMR deactivation during memory retrieval. The outlined findings suggest that the E/R flips in the PMR and right aIC represent central and interrelated sub-aspects of the neural mechanisms underlying AM performance. Building upon these findings, the present study aimed to investigate age-related changes in the E/R flips of the PMR and the aIC. For this purpose, we adopted the same incidental learning paradigm as in our previous study (Caviezel et al., 2020). The investigation of aging effects in incidental learning has at least two important advantages. First, incidental learning is assumed to be rather independent of working memory (WM) capacity (Frensch and Miner, 1994; Unsworth and Engle, 2005). As WM capacity is decreased in older subjects with and without dementia (Belleville et al., 1996; Gazzaley and Nobre, 2012), incidental learning allows investigating age-related alterations of neural memory functions independent of WM or more general cognitive deficits. Furthermore, incidental memory tasks can be assumed to include moderate or low subjective performance pressure, which under explicit learning instructions can significantly impede performance (Howard and Howard, 2001), especially in older subjects who frequently have low self-efficacy expectations (Wetherell et al., 2002; Mcdougall and Kang, 2003; Eysenck et al., 2007).

The main focus of the present study is to investigate the impact of age on E/R flips, aiming to uncover potential age-related aberrations in central modulatory neurocognitive processes. Our particular interest centered around the PMR and the aIC, which have been associated in previous fMRI investigations with underlying neurofunctional mechanisms of Am. Building upon our previous study (Caviezel et al., 2020), we utilized the same experimental setup and introduced an age-group comparison (young vs. older subjects) to identify anticipated neurofunctional changes associated with aging.

## 2 Methods

In the present study, we augmented the experimental setting of a recent study of our work group (Caviezel et al., 2020), which investigated the neural mechanisms of AM in young and healthy subjects. More specifically, we additionally investigated a group of older subjects, undergoing the same experimental procedure as the young before, in order to define age-related differences in the previously defined neurofunctional activation patterns. The study procedure was approved by the local ethics committee (Ethikkommission Nordwest- und Zentralschweiz EKNZ) and conducted in accordance with the Declaration of Helsinki.

### 2.1 Participants

In total, 45 healthy subjects participated in our study, who according to their age were divided into two sub-groups, young and older subjects. The “young group” included 23 healthy subjects, each younger than 30 years (age: mean (*m*) = 23.39 years (y), standard deviation (*sd*) = 3.23 y; 7 female; 21 right-handed; school years: *m* = 13.74 y, *sd* = 3.12 y) (Caviezel et al., 2020). The “old group” included 22 subjects, each older than 60 years (age: *m* = 69.05 y, *sd* = 4.95 y; 11 female; 20 right-handed; school years: *m* = 12.5 y, *sd* = 3.10 y). All participants were native German speakers, had normal or corrected-to-normal visual acuity, sufficient hearing abilities and reported no personal or first-degree relative history of psychiatric disorders. Prior to the imaging phase of the present study, all participants have been screened for mild cognitive impairments and signs of dementia, i.e., Alzheimer’s Dementia, using the Mini Mental State Examination (MMSE) to control for effects caused by neurodegeneration. Furthermore, to mitigate the confounding effects of sex and handedness, all participants were randomly assigned to each group based on these variables. Participants gave their written informed consent prior to their participation and received either participation hours (university course credits) or a financial incentive as compensation for their time spent. Based on the number of participants a post-hoc power analysis was conducted using G*Power to ensure the validity of the present study. The power calculation was done using an alpha error of 0.05 (two-sided), the above N per group (young: n = 23; old: n = 22) and a Cohen’s d of 1.17, which was based on the comparison of retrieval performance accuracy between young and old participants. The resulting Power (1-β) of 0.969 can be interpreted as sufficient, so that the probability of a type-2 error occurrence can be regarded as low.

### 2.2 Task

In the incidental-learning task paradigm, subjects were presented with emotionally neutral face stimuli accompanied by spoken names. Thereby, we systematically varied the contingency relation between the presented face-name combinations, which provided two experimental conditions (*cf.* Caviezel et al., 2020). In the contingency condition, face stimuli were repeatedly presented with the same name. These contingent face-name pairings provided the experimental learning condition, as they allowed subjects to associate each face with a unique name. In the non-contingency condition, on the other hand, the face-name combinations were shuffled in a controlled manner so that each face was accompanied by each name equally often (avoiding sex mismatches). These varying face-name combinations provided the no-memory baseline condition, as subjects were not able to establish specific face-name associations. At the same time, these face-name combinations provided an accurate control condition for the memory-independent sensory, cognitive and motor demands of the learning condition.

To ensure proper incidental learning conditions, subjects were instructed to give a subjective fit rating on each presented face-name combination (left button press for good fit and right button press for bad fit) but were not informed about the varying contingency relations between the stimuli. In the subsequent unannounced retrieval task, faces and names of the contingency condition were presented in varying combinations, partly violating the previously learned face-name associations. Subjects were asked to indicate whether the presented stimulus combination corresponded to the previously established association (left button press) or not (right button press) (Caviezel et al., 2020). The reason for only presenting face-name combinations of the contingency condition was that only here incidental learning had occurred.

As a no-memory retrieval condition, the same face stimuli were presented together with the word “man” or “woman,” which, spoken by the same voice, replaced the name words in the stimulus compounds of the memory condition. Thereby, subjects had to indicate whether the denoted gender matched with the presented face or not. This condition served as baseline condition and did not require any recall or other mnemonic processing. It accurately controlled for all relevant memory-independent (sensory, cognitive, and motor) processes required in the retrieval condition.

**Figure 1:**
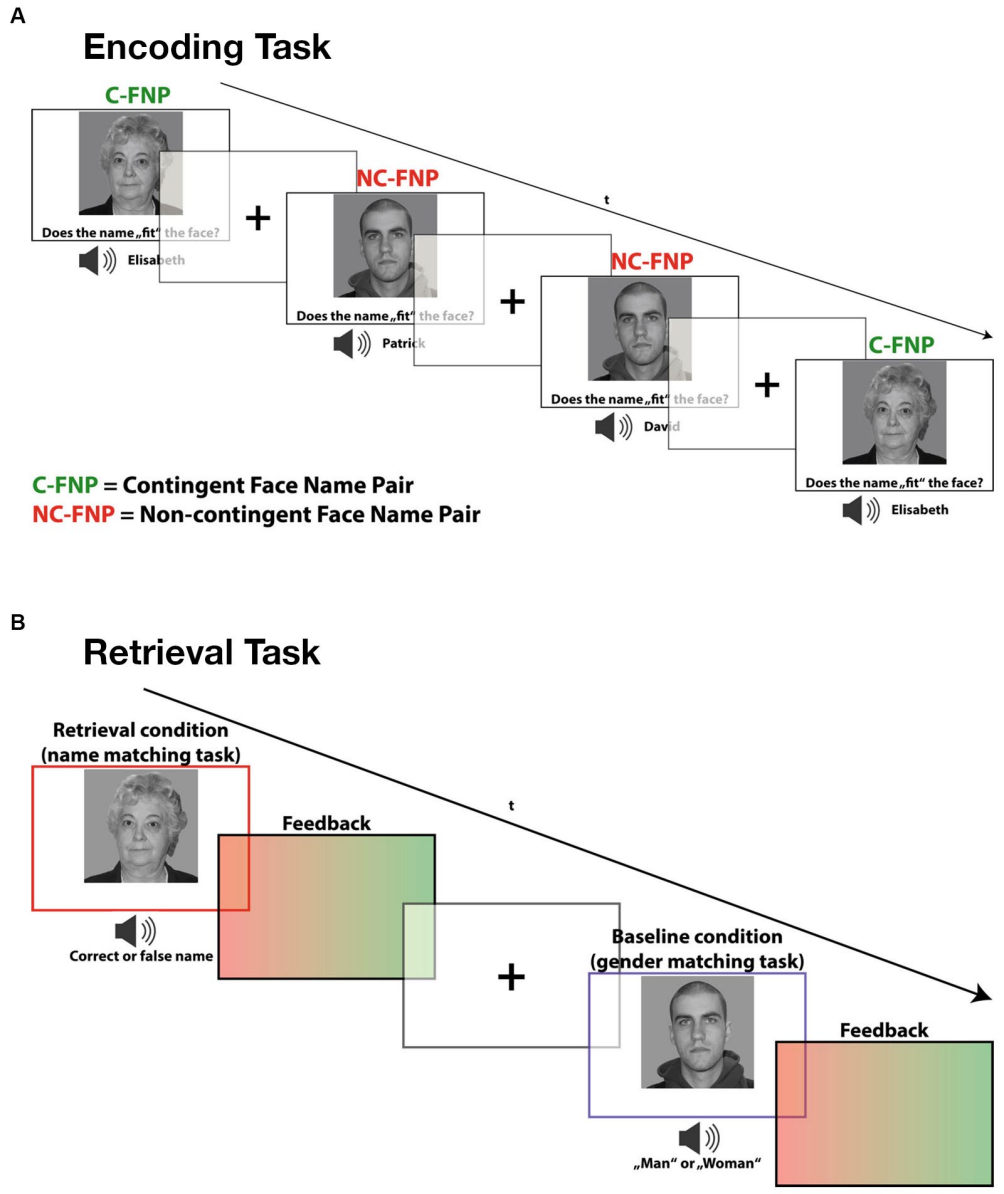
Depiction of both the encoding and retrieval task paradigm used in this study. **(A)** During encoding, participants had to learn face-name combinations and judge whether the name was part of a contingent face-name pair (Contingent) or if it was a non-contingent face-name pair (non-Contingent). **(B)** During the incidental retrieval task, participants were asked to recall the learned face-name pairs. Additionally, a gender-matching task served as a baseline condition during the retrieval task in which participants simply had to indicate whether the presented picture was male or female.

After each task trial, participants received a short (300 ms) visual feedback, consisting of a green patch indicating correct responses and a red patch for incorrect ones. In a post-experimental interview, all participants denied the use of explicit learning strategies during encoding or the expectation of a retrieval task confirming the incidental, non-intentional character of the learning processes (see Figure 1).

### 2.3 Stimuli

Twelve different faces with emotionally neutral expressions, provided by the life span database of adult facial stimuli (Minear and Park, 2004; Kennedy et al., 2009), were used and evenly distributed to the contingency and non-contingency conditions. The authors permitted us to use and adapt the provided pictures for the purpose of the present study. Faces were counterbalanced for sex and age, both across and within the task conditions. More specifically, the stimulus set comprised six male and six female faces, while each gender group included three young (*m* = 19.83 y, *sd* = 1.17 y) and three old (*m* = 66.00 y, *sd* = 3.46 y) faces. During the learning task, each face stimulus was presented twelve times for 2,800 ms resulting in a total of 144 trials (72 contingent and 72 non-contingent face-name combinations). The trail sequence was randomized, but counterbalanced for both n-1 condition transition and response side (left and right button presses). Between the target stimuli, a varying jitter interval of 500–900 ms presenting a centered fixation cross was inserted to optimize event separation and the hemodynamic response estimation. Faces were displayed in a white-black format and a 22° visual angle using a mirror system implemented in the MR head coil and a projector screen which was placed at the end of the scanner tube.

Simultaneously with the faces, auditory gender-matched name stimuli were presented, spoken by a neutral artificial male voice. The presented names were taken from the most common names in the German-speaking part of Switzerland (according to the Federal Statistical Office).^1^

The described task and stimulus presentation were implemented in E-Prime (Version 2.0, Psychology Software Tools, Pittsburgh, PA, United States) using a push-button panel and two-sided headphones.

### 2.4 Behavioral data analysis

The acquired performance data consisted of the percentages of good fit ratings for each of the two encoding conditions (for the contingent vs. non-contingent trials), the response accuracy (percentage of correct answers) and averaged response times (RT) for each task condition during retrieval. For statistical analyses, we used the software package IBM SPSS Statistics, version 27.

### 2.5 Image acquisition and data analysis

Imaging was performed in a 3 T whole-body scanner (Magnetom Prisma, Siemens Healthcare, Erlangen, Germany) using a 20-channel receive head coil.

T1 structural data were obtained using a three-dimensional (3D) magnetization-prepared rapid gradient-echo (MP-RAGE) pulse sequence (Mugler and Brookeman, 1990) with an isotropic spatial resolution of 1 mm (176 sagittal slices; time of repetition (TR) = 2000 ms; echo time (TE) = 3.37 ms; flip angle (FA) = 8°; field of view (FOV) = 256 × 256 × 176 mm^3^).

For the functional imaging data, T2*-weighted images were acquired using a blood-oxygen-level-dependent (BOLD) sensitive, interleaved gradient-echo planar imaging (EPI) sequence with a spatial resolution of 3 × 3 mm^2^ (slice thickness: 3.0 mm, 39 transversal slices; TR = 2,500 ms; TE = 30 ms; FA = 82°; FOV = 228 × 228 mm^2^).

### 2.6 Pre-processing and GLM specification

Data analysis was performed with SPM12.^2^ All volumes (i.e., EPI images) were realigned to the first volume, coregistered to the anatomical volume, normalized to the MNI305 T1 template, and finally smoothed with a 6 mm (FWHM) isotropic Gaussian kernel. During model specification, onset times for each trial of the experimental conditions were convolved with a canonical hemodynamic response (HPR) function (event-related design). Serial correlations were removed with a first-order autoregressive model, and a high-pass filter (128 s) was applied to remove low-frequency noise. To control for potential head motion, we plotted the head motion parameters of each individual and visually screened whether there were any outliers greater than a voxel size, i.e., 2 mm or 2° in translation or rotation. In all participants, head motion was less than 2 mm, and translation and rotation were less than 2° in any direction. Therefore, we did not conduct any head motion corrections. Nevertheless, the six movement parameters were included as nuisance covariates in the GLM of first-level analyses.

### 2.7 fMRI data analyses

To reveal encoding-related brain activations at the single-subject level, the contingency condition was contrasted against the non-contingency condition. Retrieval-related activations, on the other hand, were defined by contrasting the trials of all successfully retrieved face-name combinations against the trials of the baseline (i.e., gender matching) condition. The thereby generated first-level contrast images were subsequently propagated to second-level analysis using one-sample t-tests (random effects models). On the second level, pairwise group comparisons (t-tests) and related conjunction analyses were applied following the minimum statistic method (null conjunction) to examine the differences and commonalities regarding the E/R flip in the young and old subject samples.

**Figure 2:**
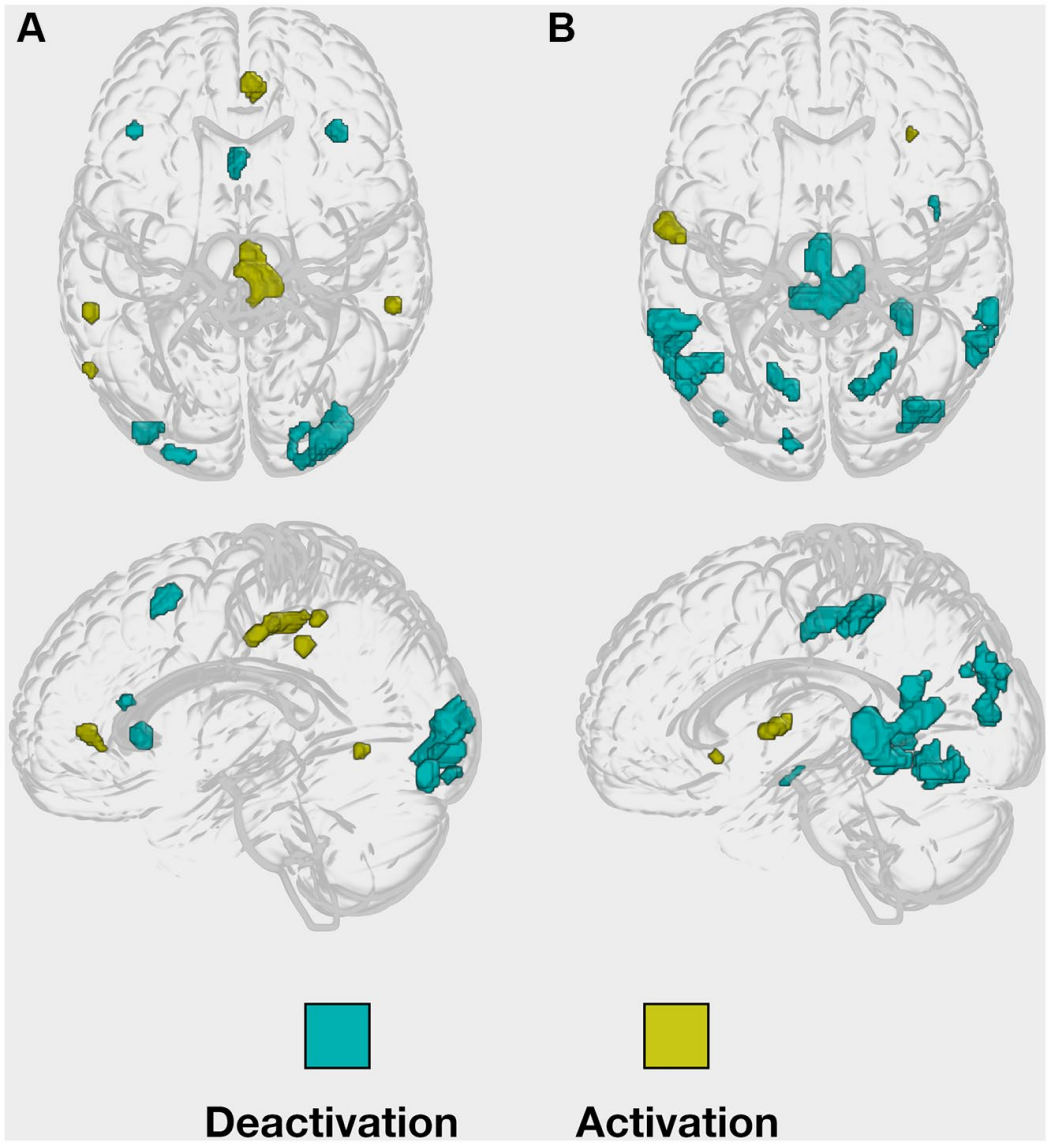
Implicit masks of whole-brain activations obtained from the young healthy group. Whole-brain (de-)activation patterns, were observed during both **(A)** the encoding and **(B)** the retrieval task.

For our hypotheses testing, on the whole-brain level, the intensity threshold of the peak voxel was set to a *p*-value of 0.001, uncorrected, and the minimal cluster size threshold k was set to a minimum of five contiguous voxels. To define age-related functional activation changes in the task-relevant regions, we compared the young with the older subject group by applying an inclusive masking procedure in order to restrict the findings to the task-relevant regions previously revealed in the young sample of Caviezel et al. (2020), see Figure 2. Accordingly, a small volume correction for the inclusive mask was applied, demanding a p-value of 0.05, familywise error (FWE) corrected, as significant intensity threshold for the peak-voxel.

### 2.8 Correlation analyses: neural activity × performance measures

Furthermore, we performed Pearson’s correlations to assess whether retrieval-related performance measures, i.e., accuracy and reaction times, were associated with neural activity, i.e., eigenvalues, in regions relevant to AM functioning during both encoding and retrieval. The correlations were calculated separately for each group. Finally, we applied the Fisher’s *Z* test to see whether correlations differed significantly between groups.

## 3 Results

### 3.1 Behavioral results

#### 3.1.1 Older subjects learned face-name combinations incidentally

In the encoding task, we found significant condition differences regarding both the proportion of response categories (fit vs. non-fit ratings) and response time. More specifically, participants rated a significantly higher percentage of face-name combinations as fit in the contingency condition than in the non-contingency condition (contingency: *m* = 67.11%, *sd =* 12.47%; non-contingency: *m* = 31.00%, *sd* = 19.82%; *t*(21) = 7.06, *p* < 0.001). Furthermore, participants gave their responses significantly faster in the contingency condition than in the non-contingency condition (contingency: *m* = 1,299 ms, *sd* = 181 ms; non-contingency: *m* = 1,508 ms, *sd* = 249 ms; *t*(21) = −5.64, *p* < 0.001).

In the retrieval task, the accuracy was high in both conditions, participants gave significantly fewer correct answers in the retrieval condition compared to the baseline condition (retrieval: *m* = 79.55%, *sd* = 2.52%; baseline: *m* = 85.35%, *sd* = 3.0%; *t*(21) = −2.376, *p* = 0.027). Moreover, participants gave their responses significantly slower in the retrieval condition than in the baseline condition (correct retrieval: *m* = 1,370 ms, *sd* = 121 ms; baseline: *m* = 1,180 ms, *sd* = 135 ms; *t*(21) = 8.249, *p* < 0.001). They also showed significantly faster RT for correct compared to incorrect responses in the retrieval condition (retrieval: *m_cor_* = 1,360 ms, *sd_cor_* = 121 ms; *m_incor_* = 1,450 ms, *sd_incor_* = 134 ms; *t*(20) = 3.87, *p* < 0.001).

#### 3.1.2 Older subjects showed age-related deficits in AM

**Figure 3:**
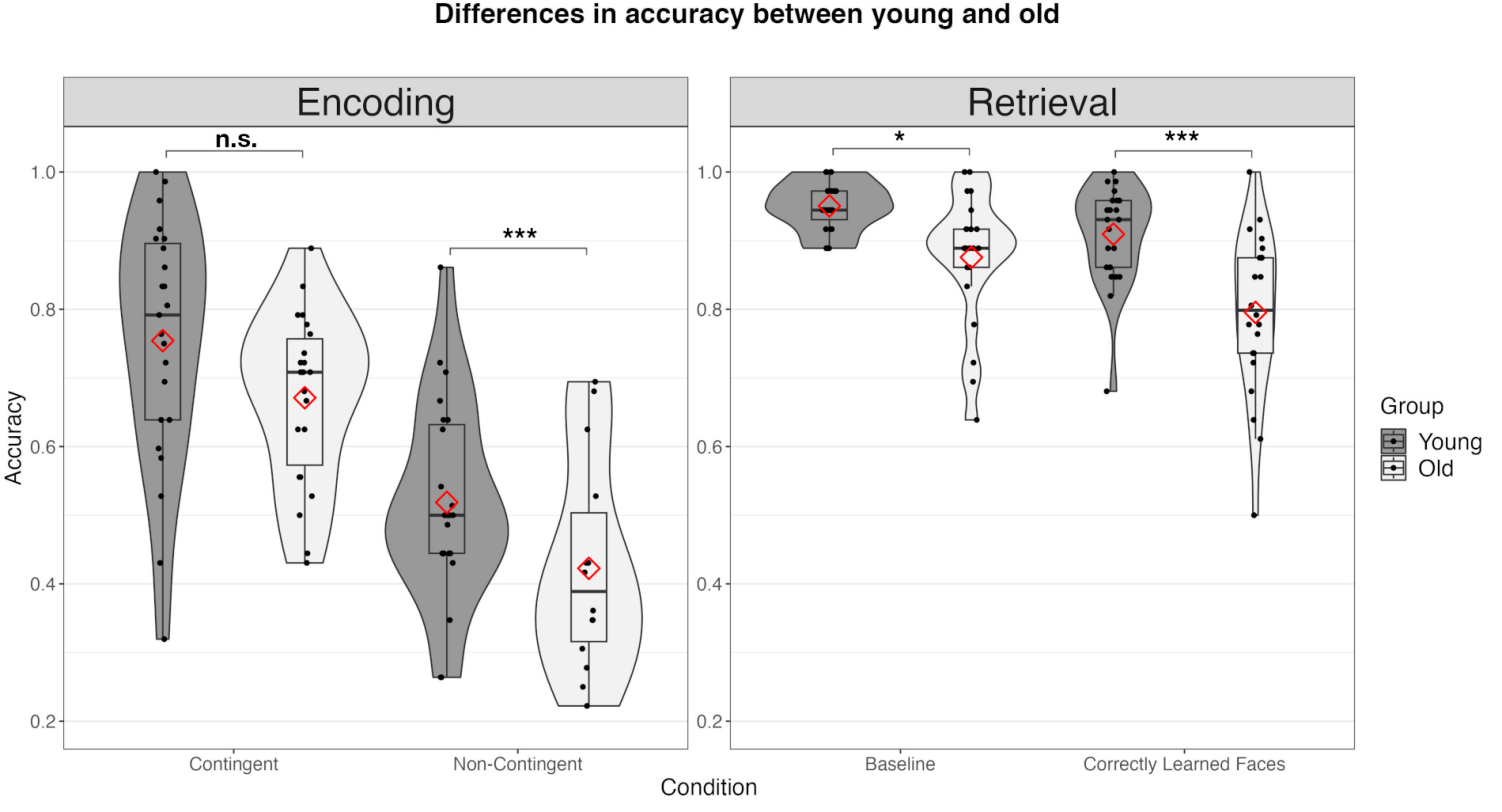
Differences in accuracy between the young and the old group across all conditions. Combined violin and boxplots represent the median, 1. and 3. interquartile range, and minimum and maximum range of performance. Red diamonds represent mean values. n.s., not significant; * = *p* < 0.05; ** = *p* < 0.01; *** = *p* < 0.001.

**Figure 4:**
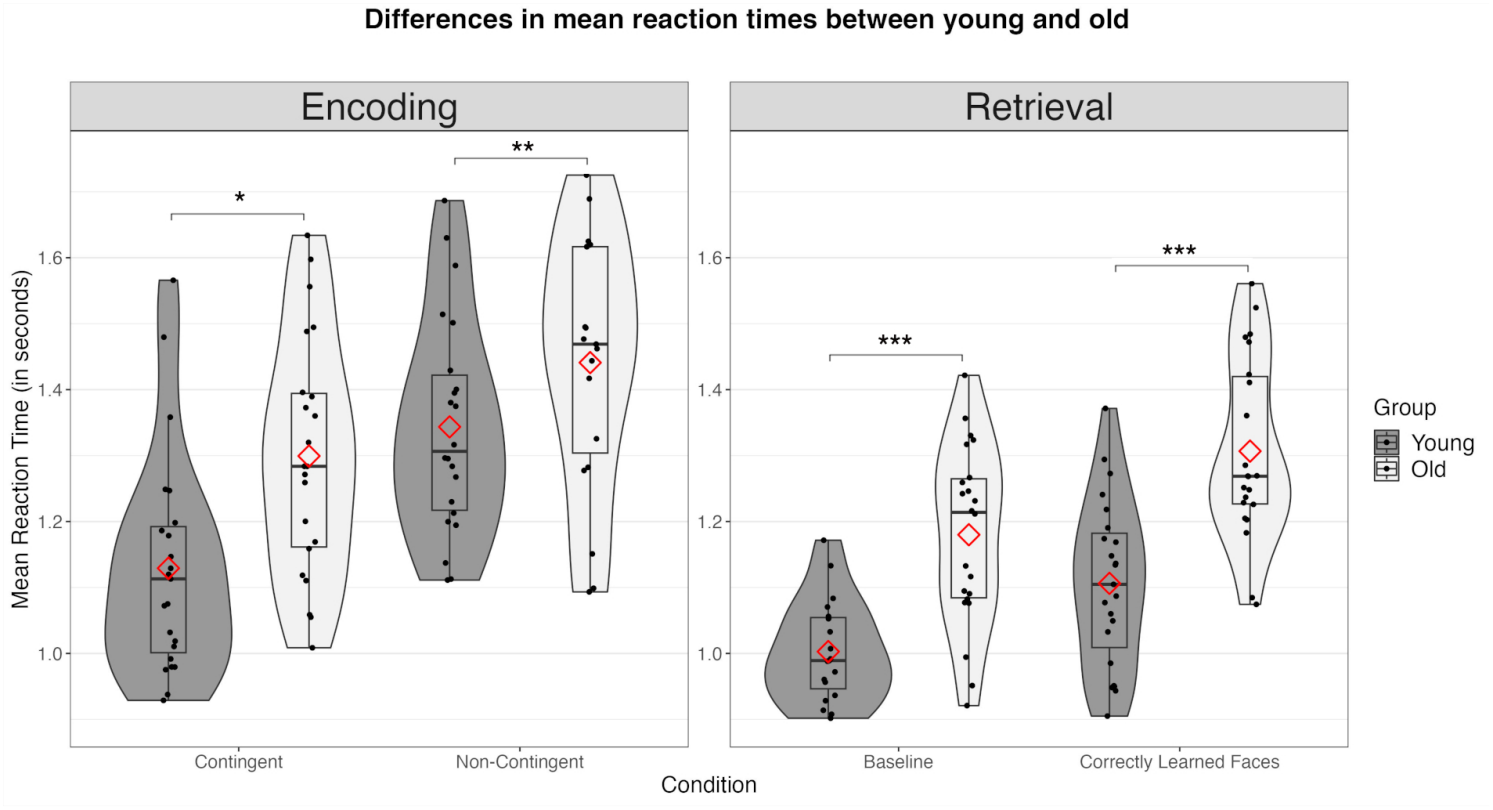
Differences in mean reaction times (in seconds) between the young and the old group across all conditions. Combined violin and boxplots represent the median (black bar), 1. and 3. interquartile range, and minimum and maximum range of performance. Red diamonds represent mean values. n.s., not significant; * = *p* < 0.05; ** = *p* < 0.01; *** = *p* < 0.001.

Comparing the behavioral data between groups, we observed significant age-related deviations across all measures. First, during encoding, older participants demonstrated a significantly reduced proportion of “good fit” ratings in the non-contingency (young: *m* = 51.87%, *sd* = 14.37%; old: *m* = 30.99%, *sd* = 19.82%; *t*(43) = −4.06, *p* < 0.001) but not in the contingency condition (young: *m* = 75.42%, *sd* = 17.87%; old: *m* = 67.1%, *sd* = 12.47%; *t*(43) = −1.80, *p* = 0.079) (see Figure 3). Older subjects exhibited significantly increased RTs in both the non-contingency (young: *m* = 1,362 ms, *sd* = 183 ms; old: *m* = 1,507, *sd* = 249 ms; *t*(43) = 2.232, *p* = 0.030) and contingency conditions (young: *m* = 1,130 ms, *sd* = 167 ms; old: 1300 ms, *sd* = 180 ms; *t*(43) = 3.276, *p* = 0.002) (see Figure 4).

Concerning memory retrieval, older subjects showed a clear decline in accuracy compared to young subjects during both the baseline (young: *m* = 95.05%, *sd* = 3.55%; old: *m* = 85.35%, sd = 14.01; *t*(34.5) = −3.15, *p* = 0.004) and the retrieval condition (old: *m* = 79.55%, *sd* = 11.80%; young: *m* = 90.94%, *sd* = 7.22%; *t*(34.5) = −3.887, *p* < 0.001) (see Figure 3). Accordingly, older subjects were significantly slower compared to the younger group (baseline: young: *m* = 972 ms, *sd* = 100 ms; old: *m* = 1,180 ms, *sd* = 135 ms; *t*(43) = 5.850, *p* < 0.001; memory retrieval: young: *m* = 1,106 ms, *sd* = 135 ms; old: *m* = 1,306 ms, *sd* = 121, *t*(43) = 5.635, *p* < 0.001) (see Figure 4).

### 3.2 fMRI imaging results

#### 3.2.1 Encoding: common and distinct activations of younger and older subjects

The conjunction analysis revealed common memory-related activations (contingency > non contingency) in two midline regions, the bilateral pMCC and the right anterior cingulate cortex as well as in the left inferior parietal (supramarginal) gyrus and the right middle temporal gyrus were activated in both groups. The group contrast revealed only one significant activation difference situated in the left middle temporal gyrus. It reached statistical significance only in the young, but not in the old subject sample. Importantly, the older subject group did not show significant encoding-related deactivations (contrast: non-contingency > contingency), whereas the younger subjects produced a prominent pattern of deactivated regions during memory encoding. This activation pattern encompassed the bilateral posterior-medial frontal gyrus, the left inferior frontal gyrus, the right inferior occipital and the left middle occipital gyrus, including the right aIC (see Table 1).

**Table 1:** Activations and deactivations observed during the encoding task in both young and old.

Anatomy	MNI coordinates x,y,z		*k*		*T_max_*		Young vs. Old					
	Young	Old	Young	Old	Young^+^	Old*	Conjunction			Young > Old		
							MNI	*k*	*T_max_*	MNI	*k*	*T_max_*
*Activation*												
Right Anterior cingulum	6, 42, 2	4, 40, 42	43	24	7.41^+^	4.49*	6, 42, 2	23	4.52*	n.s.		
Posterior middle cingulate cortex (bilateral)	4, −18, 38	4, −30, 42	149	79	10.03^+^	5.56*	2, −18, 38	90	6.34*	n.s.		
Left supramarginal gyrus	−58, −42, 42	−54, −42, 40	27	14	6.86^+^	4.19*	−56, −46, 42	8	3.94*	n.s.		
Left middle temporal gyrus	−56, −64, 0	n.s.	13	n.s.	6.76+	n.s.	n.s.			−56, −64, 2	1	3.35
Right middle temporal gyrus	58, −38, 38	54, −40, 42	20	20	5.42^+^	6.08*	54, −42, 40	11	4.34*	n.s.		
*Deactivation*												
Posterior-medial frontal gyrus (bilateral)	0, 16, 52	n.s.	55	n.s.	6.62^+^	n.s.	n.s.			0,18, 50	32	4.20*
Right anterior insular cortex	36, 24, −2	n.s.	47	n.s.	7.78^+^	n.s.	n.s.			34, 26, −2	47	5.50*
Left inferior frontal gyrus (p. triangularis)	−42, 26, 20	n.s.	16	n.s.	5.19^+^	n.s.	n.s.			−40, 26, 18	16	4.42*
Left middle occipital gyrus	−40, −84, −8	n.s.	71	n.s.	6.26+	n.s.	n.s.			−30, −84, −8	29	4.33*
Right inferior occipital gyrus	30, −92, −2	n.s.	390	n.s.	8.77+	n.s.	n.s.			32, −88, 6	226	5.44*
Left middle occipital gyrus	−20, −96, −8	n.s.	32	n.s.	6.57+	n.s.	n.s.			−30, −84, −8	29	4.33*

^+^*p_peak_* < 0.05, Family-wise error (FWE)-corrected on peak level; *p_Peak_ < 0.05, small volume corrected and FWE-corrected on peak level; *k* = cluster size; *T*_*Max*_ = maximum *t*-value at peak voxel.

#### 3.2.2 Retrieval: common and distinct activations of younger and older subjects

During memory retrieval (contrast: retrieval > baseline), there was only one region of significant common activation for young and older subjects, located in the left superior temporal gyrus. Important to note, the right aIC – which in our previous study exhibited a prominent E/R flip in young subjects - was not significantly activated in the older subject group.

Common retrieval-related deactivations, (contrast: baseline > retrieval) exhibited significant commonalities regarding retrieval-related deactivations comprising of the pMCC, right fusiform gyrus, bilateral middle posterior temporal gyrus and left lingual gyrus (FWE and small volume corrected). Other regions, particularly in the occipital cortex, such as the right lingual gyrus, right middle occipital gyrus and left cuneus demonstrated functional de-activation only in the younger but not in the older subjects. Interestingly, only the right middle occipital gyrus, demonstrated a significant age effect during retrieval-related deactivation (see, Table 2).

**Table 2:** Activations and deactivations observed during the retrieval task in both young and old.

Anatomy	MNI coordinates x,y,z		*k*		*T_max_*		Young vs. Old					
	Young	Old	Young	Old	Young	Old	Conjunction			Young > Old		
							MNI	*k*	*T_max_*	MNI	*k*	*T_max_*
*Activation*												
Right anterior insular cortex	32, 26, -6	n.s.	8	n.s.	4.97^+^	n.s.	n.s.			36, 28, −8	7	4.01
Left superior temporal gyrus	−58, −14, 0	−60, −8, 0	61	61	7.10^+^	9.24*	−60, −8, 0	61	6.78*	n.s.		
*Deactivation*												
Posterior middle cingulate cortex (bilateral)	−2, −20, 44	−12, −34, 42	337	143	7.91^+^	5.49*	6, −30, 46	143	5.32*	n.s.		
Right fusiform gyrus	28, −42, −10	−30, −50-10	67	41	5.62^+^	4.83*	30,-50,-10	41	4.83*	n.s.		
Left posterior middle temporal gyrus	−58, −46, 2	−62, −48, −6	574	128	8.33^+^	4.85*	−62, −48, −6	128	4.85*	n.s.		
Right middle posterior temporal gyrus/Inferior temporal gyrus	54, −54, −8	62, −50, −4	127	36	8.68^+^	4.40*	62, −50, −4	36	4.40*	n.s.		
Right lingual gyrus	14, −68, −4	n.s.	101	n.s.	5.22^+^	n.s.	n.s.			16, −70, −16	3	3.26
Left lingual gyrus	−12, −72, −14	−12, −70, −10	57	n.s.	5.10^+^	n.s.	−12, −70, −10	6	3.60	n.s.		
Right middle occipital gyrus	42, −78, 10	n.s.	222	n.s.	7.25^+^	n.s.	n.s.			42, −78, 10	40	4.69*
Left Cuneus	−12, −92, 24	n.s.	25	n.s.	6.25^+^	n.s.	n.s.			−12, −92, 26	3	3.50

^+^*p_peak_* < 0.05, Family-wise error (FWE)-corrected on peak level; *p_Peak_ < 0.05, small volume corrected and FWE-corrected on peak level; *k* = cluster size; *T*_*Max*_ = maximum *t*-value at peak voxel.

#### 3.2.3 The E/R flip in the right aIC compared between age groups

**Figure 5:**
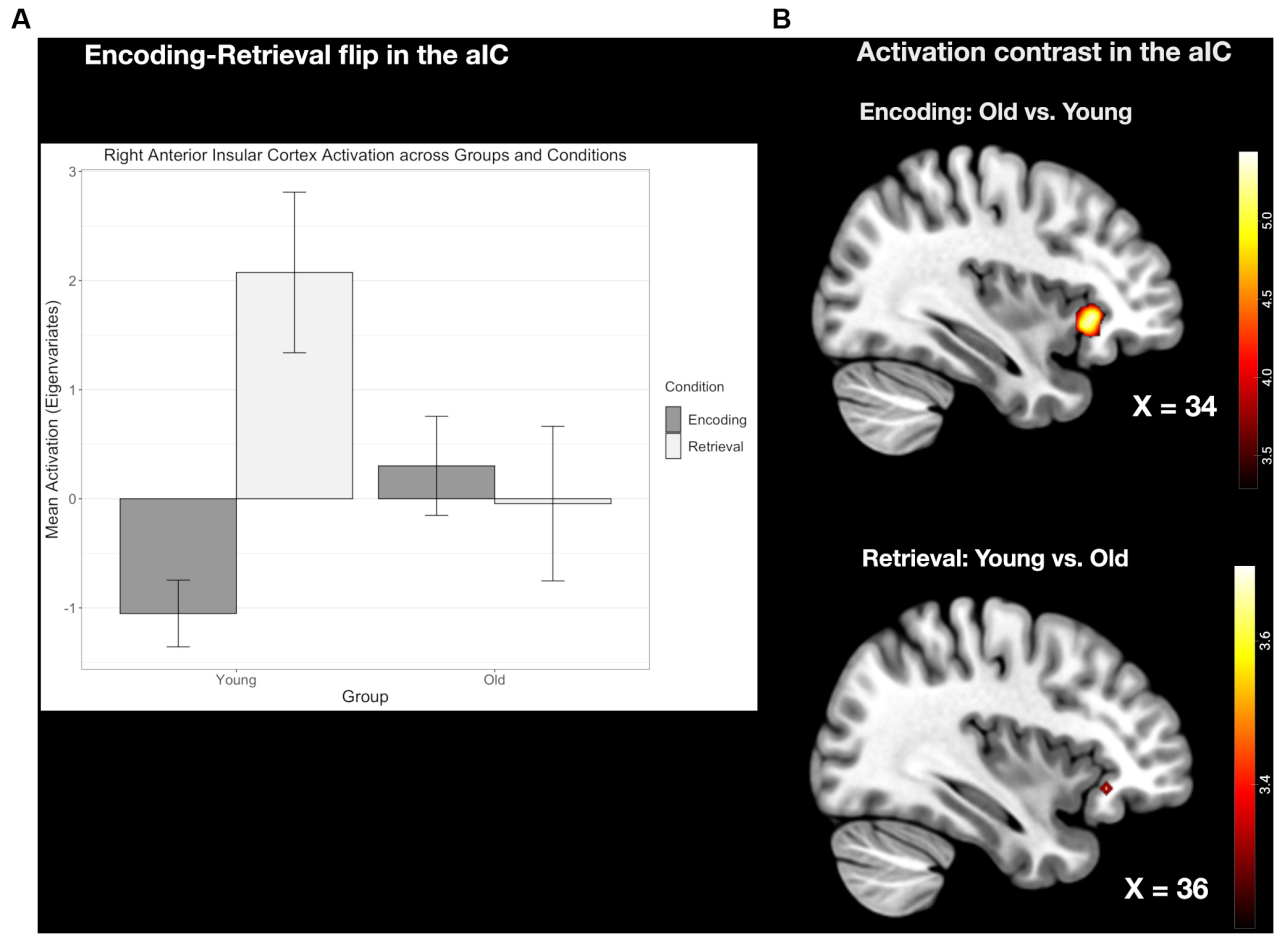
**(A)** Contrasting activation within the right aIC during encoding and retrieval, respectively. Error bars represent standard deviations from the mean. **(B)** Clustered bar plot demonstrating an absence of the Encoding/Retrieval flip in the old group compared to the young group.

In the young sample, a prominent E/R flip was found in the right aIC, exhibiting a deactivation during encoding and an activation during retrieval. In the older sample, this effect was completely absent. Instead, older subjects exhibited neither significant deactivation during encoding nor significant activation during retrieval in the right aIC. A direct group comparison revealed a significant difference in the deactivation during encoding and also in the activation during retrieval, while the corresponding focus, however, lay slightly outside the applied mask of aIC activation in the young subject group (x = 36, y = 28, z = −8, *k* = 7, *T_max_* = 4.01, *p* < 0.001 uncorrected at whole brain level; see Figure 5).

#### 3.2.4 The E/R flip in the right pMCC compared between age groups

**Figure 6:**
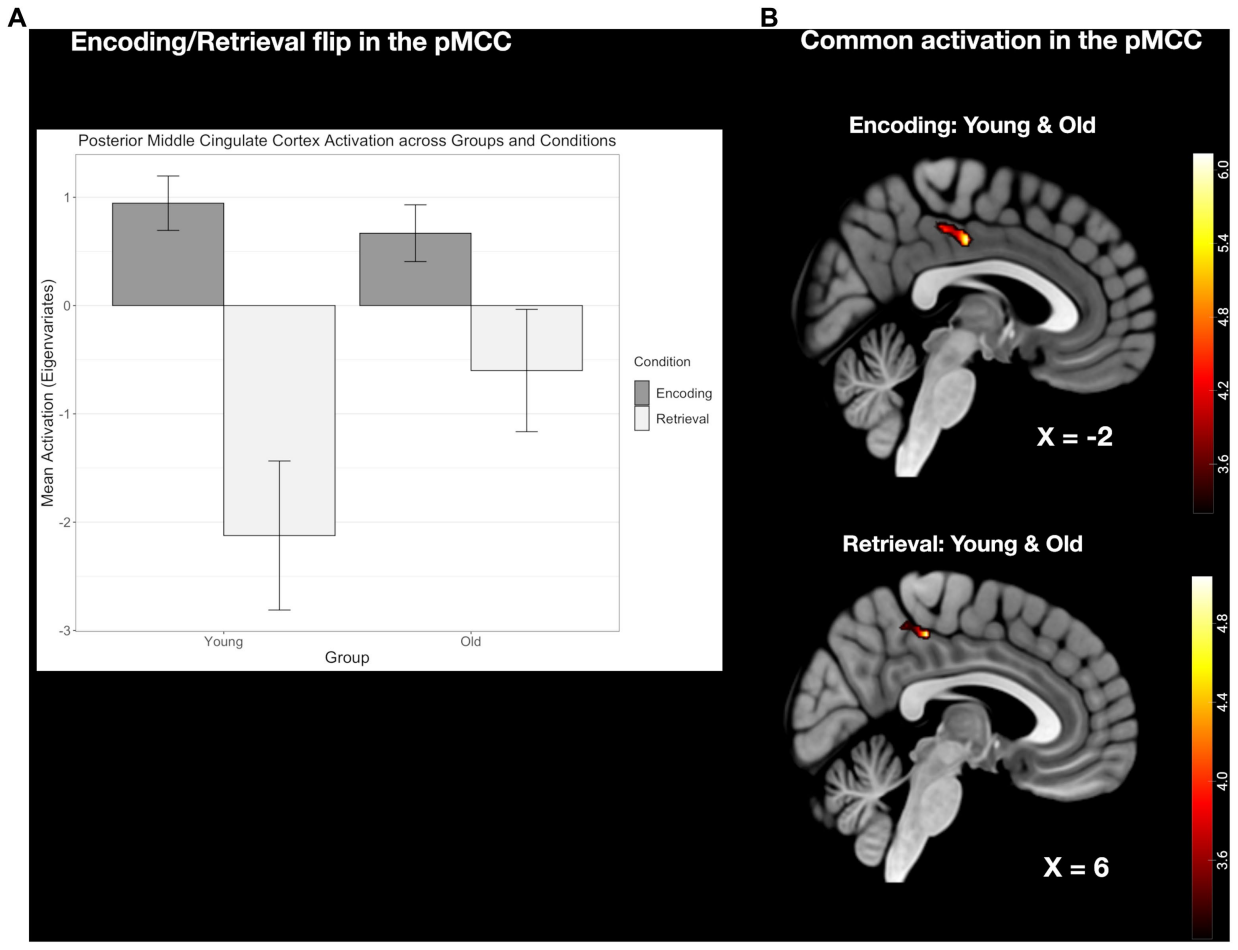
**(A)** Clustered bar plot indicating equivalent and significant encoding-related activation and retrieval-related deactivation patterns in the pMCC across both groups. Error bars represent standard deviations from the mean. n.s., not significant. **(B)** Common activation in the pMCC found in both the young and old group during the encoding and retrieval task.

The conjunction analyses revealed, common activation during encoding and common deactivation during retrieval in the same region of the pMCC in the two age groups. This common E/R flip, however, was reduced in its overall activation magnitude in the older subject group stemming both from a reduced activation during encoding and reduced deactivation during retrieval. Although both age groups exhibited a prominent E/R flip in the pMCC, the overall activation magnitude was reduced in the older subject group in both conditions (see Figure 6). However, these activation differences did not reach the level of significance.

#### 3.2.5 The E/R flip in the left MTG compared between age groups

**Figure 7:**
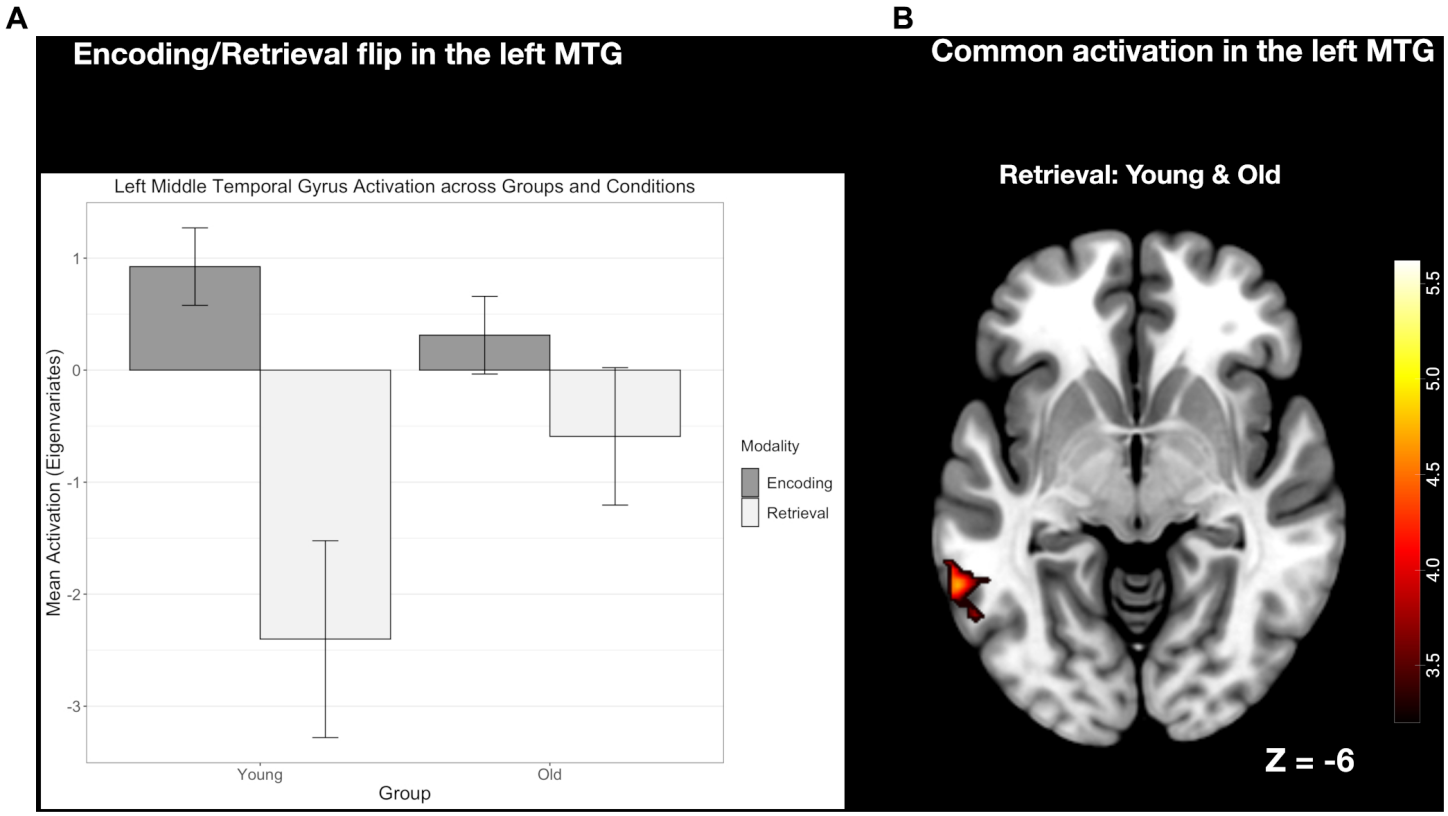
**(A)** Clustered bar plot showing equivalent and significant encoding-related activation and retrieval-related deactivation patterns in this region across both groups. Error bars represent standard deviations from the mean. n.s., not significant. **(B)** Common activation in the left MTG found during retrieval but not during encoding.

For the left MTG, which exhibited an E/R flip in the young group, old subjects did not reveal an activation during encoding but a common deactivation for retrieval. At the descriptive level, the de-activation during retrieval appeared to be reduced in the old age group, while the corresponding group difference, again, did not reach statistical significance (see Figure 7).

#### 3.2.6 Age differences in the correlations between E/R flip related brain (de-)activations and memory performance

##### 3.2.6.1 Performance accuracy

**Figure 8:**
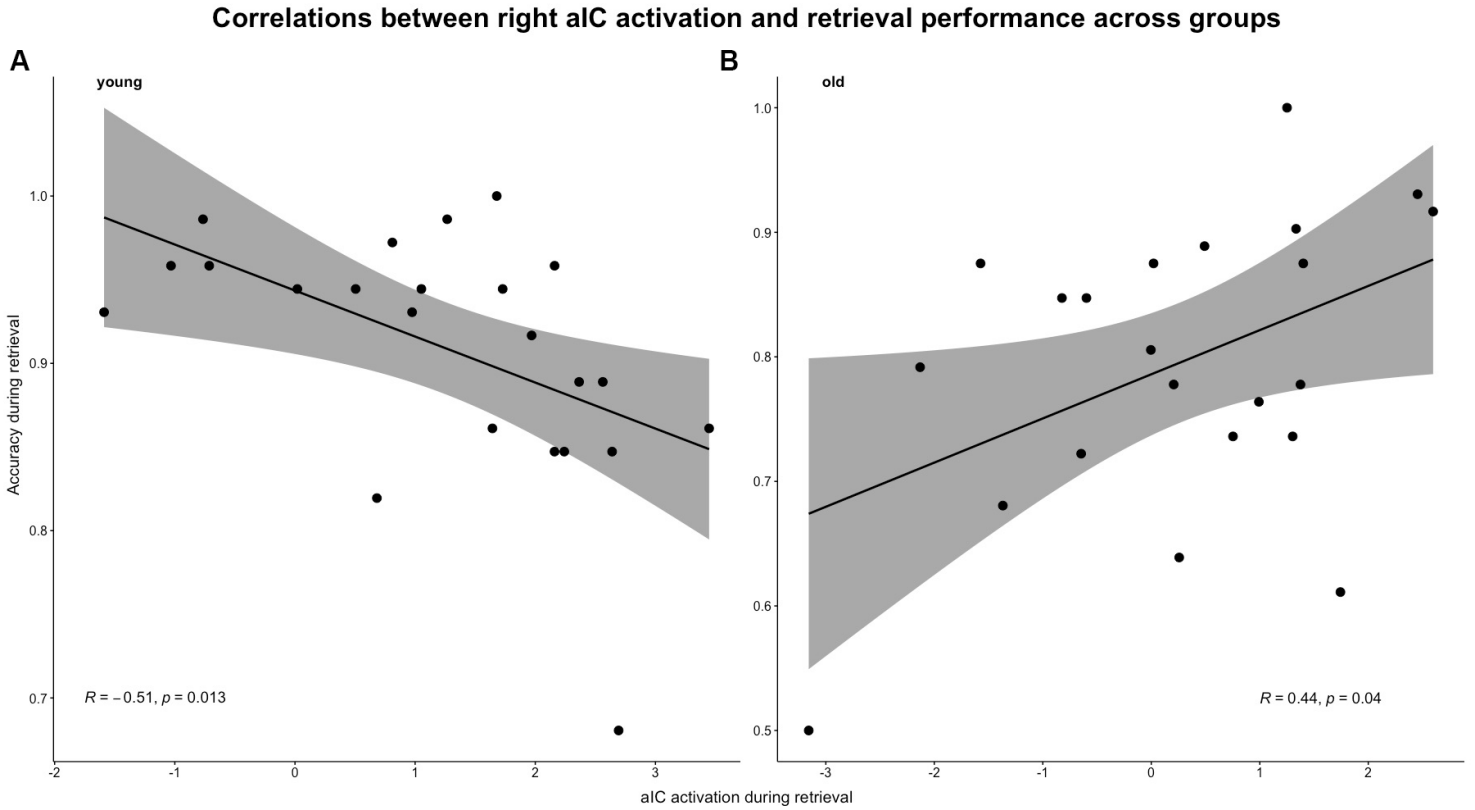
Pearson’s correlations between aIC activation and task-related accuracy during the retrieval condition. Correlations were calculated separately for both groups: **(A)** young group and **(B)** old group.

Even though correlations between the encoding-related aIC deactivation and performance accuracy did not reach significance in both groups (young: *r* = 0.243, *p* = 0.264; old: *r* = 0.172, *p* = 0.443), the correlations between retrieval-related activation strength in the aIC and performance accuracy reached significance in both groups (young: *r* = −0.510, *p* = 0.012; old: *r* = 0.442, *p* = 0.039) (for reference, see Figure 8). The Fisher’s *z*-test confirmed that the correlations differed significantly between age groups (*z* = − 2.339, *p* = 0.01, *q* = −0.749).

The correlations between the pMCC’s encoding-related activation and performance accuracy did not surpass the significance threshold of *p* = 0.05 in both the young (*r* = 0.323, *p* = 0.133), and older subjects (*r* = −0.246, *p* = 0.270). During retrieval, negative correlation between the pMCC’s retrieval-related deactivation and performance accuracy was found in the young (*r* = −0.322, *p* = 0.067), and old group (*r* = −0.015, *p* = 0.473). Both correlations were not significant but reached statistical trend level for the younger subject group.

The correlations between MTG activation during encoding and performance accuracy remained insignificant in both groups (young: *r* = 0.277; *p* = 0.20; old: *r* = −0.059; *p* = 0.793). However, the correlations between the retrieval-related deactivation and performance accuracy revealed a positive trend for the young (*r* = 0.388; *p* = 0.067) but not the old group (*r* = −0.251; *p* = 0.26).

##### 3.2.6.2 Reaction times

The correlations between encoding-related aIC deactivation and reaction times did not reach significance in both groups (young: *r* = −0.064, *p* = 0.773; old: *r* = −0.404, *p* = 0.062). Similarly, the correlations between reaction times and retrieval-related aIC activation did not reach significance in both age groups (young: *r* = 0.023, *p* = 0.917; old: *r* = −0.293, *p* = 0.185).

In the pMCC, correlations between encoding-related pMCC activation and reaction times did not reach significance in either group (young: *r* = −0.086, *p* = 0.698; old: *r* = 0.229, *p* = 0.306). However, the correlations between retrieval-related deactivation and reaction times did reach significance in the young group (young: *r* = 0.464, *p* = 0.026) but not in the old group (old: *r* = −0.003, *p* = 0.99).

Finally, the correlations between the encoding-related activation in the MTG and reaction times were not significant (young: *r* = 0.153, *p* = 0.486; old: *r* = −0.035, *p* = 0.877). Also, the correlations between retrieval-related MTG deactivation and reaction times did not reach significance in both groups (young: *r* = 0.184, *p* = 0.401; old: *r* = 0.273, *p* = 0.220).

## 4 Discussion

In the present study, we investigated age-related changes in the neural mechanisms underlying AM performance during both mnemonic encoding and retrieval by augmenting the data set of a previous study in young healthy subjects (Caviezel et al., 2020) by a sample of older subjects. We specifically focused on potential age-related changes in so-called E/R flips, which are characterized by an inverse pattern of activation and deactivation in circumscribed brain regions during encoding and retrieval. For this purpose, we designed a memory task paradigm which specifically addresses incidental, i.e., non-intentional, rather than explicit, thus intentional, learning. The reason for this decision is that incidental, unlike explicit, learning has been shown to be widely independent of working memory capacity (Unsworth and Engle, 2005). Therefore, this paradigm arguably allows to define age-related changes in AM widely independent of WM deficits, which occur in the course of aging (Ellis, 2009) and may underlie putative deficits in AM functioning (Bender and Raz, 2012).

The current results basically confirm our initial hypothesis, as we observed age-related changes in the E/R flips, especially concerning the E/R flip in the aIC. More specifically, the present study demonstrates a clear and specific age-related decline in the neural mechanisms underlying AM functioning, which is reflected in three main findings. First, on a behavioral level, older subjects demonstrated a significantly reduced memory performance including both accuracy and reaction times during mnemonic encoding and retrieval. Second, older subjects exhibited distinct reductions in the E/R flips of the posterior middle cingulate cortex (pMCC), and most prominently the anterior insular cortex (aIC), which have been corroborated as central neurofunctional regions that importantly underlie AM functioning. Third and last, younger and older subjects exhibited opposed correlations between performance accuracy and retrieval-related functional (de-)activations, which was observed in the aIC. The outlined findings are discussed in detail below.

### 4.1 Behavioral findings confirm performance-related deficits in aged individuals

During encoding, both groups showed faster response times and higher accuracy in the contingency compared to the non-contingency condition, which basically confirmed the occurrence of incidental learning in both age groups. Likewise, during retrieval both age groups showed a reduced response accuracy and reaction time in the memory compared to the non-mnemonic baseline, i.e., gender matching, condition. Taken together, the current behavioral findings confirm the occurrence of the intended memory processes in both groups, while the conducted group comparisons confirmed a broad age-related performance decline which became evident across all performance measures.

These findings follow a previous line of evidence showing the AM to be among the first cognitive functions declining with age (Naveh-Benjamin and Mayr, 2018), including *incidental* association learning (e.g., Eysenck, 1974; Mitchell, 1989; Plancher et al., 2010). However, these previous studies mainly investigated free recall of incidentally learned information and not recognition as we did. Following their comprehensive meta-analysis, Old and Naveh-Benjamin (2008) argued for a gradient of differential susceptibility to age-related decline in different kinds of AM performance. Thereby, age-related AM loss was most pronounced in free recall, diminished in a cued recall and absent in recognition. It is further postulated that incidental learning is mainly impaired in high demanding retrieval tasks such as free recall and when deep compared to shallow encoding of the information is demanded (Wagnon et al., 2019). Our behavioral findings clearly contradict these well-grounded theoretical notions in central aspects. A reason why we found an age-related decline for mnemonic recognition may lie in the use of names as memory items, which are rather unspecific (i.e., relate to different people and episodes of our live history) and therefore are more susceptible to intrusions. The susceptibility to intrusions has also been described to increase with age (Kahana et al., 2005), so that intrusions presumably evoked by the presented name items may account for the observed age-related memory performance deficit in the recognition task.

### 4.2 The E/R flip in the aIC is an important marker for age-related mnemonic memory decline

The present study’s findings indicate that the E/R flip in aIC, while still intact in the young subjects, was highly diminished, if not completely absent, in the older subjects. This prominent role of the aIC is well in line with previous neuroimaging studies which could relate abnormal insula function to age-related episodic memory decline (Xie et al., 2012; Muller et al., 2016). More generally, the aIC has been shown to basically underlie interoception, including both body representation and emotional experience (Seeley et al., 2007). Furthermore, the aIC has been described as central hub of the salience network (SN), responding to relevant events or stimuli, even if they fall outside the current focus of attention (Muller et al., 2016; Ghaziri et al., 2017; Seeley, 2019). Moreover, the aIC has been corroborated as central neural switching instance that adaptively mediates between engagement and disengagement of different functional networks. For instance, it has been shown to coordinate the interaction between the default mode network (DMN) and the central executive network (CEN) that is responsible for goal-directed information processing, decision-making, and behavioral control (Corbetta and Shulman, 2002; Melcher and Gruber, 2006; Sridharan et al., 2008; Ghaziri et al., 2017; Uddin et al., 2017). In advanced age, the function of the DMN is often altered or impaired, which specifically concerns the ability to deactivate the DMN during cognitively demanding tasks (Grady et al., 2006; Persson et al., 2007; Meinzer et al., 2012). This declined deactivation of the DMN can be ascribed to an impaired regulatory function of the insular cortex (Muller et al., 2016), which accordingly may be more closely related to age-related memory decline than regions within the DMN. Generally, brain-functional and structural alterations in insula networks have the potential to impair older adults’ cognitive performance in many respects, including mnemonic abilities comprising both encoding and retrieval (Xie et al., 2012). Thus, the aberrant E/R flip in the aIC in the present study can be considered an important marker of neurocognitive aging, underlying specific declines in AM performance and also other cognitive domains in older subjects.

### 4.3 The E/R flip in the PMR did not exhibit an age effect

Our current findings revealed a joint E/R flip in the pMCC in both age groups. Furthermore, the E/R flip in the pMCC exhibited a decreased activation strength during both encoding and retrieval in the old subjects compared to the young subjects. Although this age-related modulation of the pMCC was insignificant following the direct age comparison, this finding is still well in line with previous studies that observed an age-related decline in pMCC modulation strength during AM. At the same time, this age-related decline in the pMCC is associated with a deterioration in behavioral performance during AM processes (Vannini et al., 2013; Amlien et al., 2018; Fu et al., 2020). Furthermore, the pMCC, as a central hub of the DMN network, shows high functional connectivity with the right aIC and, more specifically, demonstrated a negative connectivity with the right aIC during retrieval in young subjects (Caviezel et al., 2020). Coinciding with the age-related reduction in modulation strength of the pMCC, the functional connectivity between right aIC and pMCC shows a marked decrease with age (Chen et al., 2018), and has been associated with reduced performance accuracy (O’Connell and Basak, 2018). Therefore, both the reduction in activation strength and in connectivity between pMCC and aIC can plausibly be attributed to the reduction in functionality of the DMN network in the beginning and advanced stages of aging.

### 4.4 “Inverse” ER-flip in pMCC and the left MTG

It is important to note, that in both groups the E/R flip pattern in the pMCC and left MTG was exactly inverted with respect to the findings of the previous E/R flip literature (*cf.* Huijbers et al., 2012); meaning that both structures were activated during encoding and deactivated during retrieval. Most likely, this alleged discrepancy is due to the differences in processing modes that are implemented during memory encoding and retrieval. Compared to previous studies on AM (Daselaar et al., 2009; Vannini et al., 2011; Huijbers et al., 2012), the present investigation operationalized encoding as a non-deliberate, incidental learning task by presenting a non-mnemonic mock task on the to-be-learned stimuli. Incidental cognitive processes bear the important advantage to be widely independent of WM deficits (Reber et al., 1991; Wagner, 1999), which could influence or override more specific neurocognitive and age-related processes (Unsworth and Engle, 2005). As WM activity is well known to decrease or even interrupt activity in the DMN and particularly in the PMR (Sambataro et al., 2010; Nussbaumer et al., 2015), we assume that the reduced demands on WM capacities are responsible for the increased functional activation in the pMCC and left MTG during incidental encoding. As both structures belong to the DMN (Damoiseaux et al., 2006; Uddin et al., 2009), their activation is not affected by our incidental encoding task. A prior study by Yang et al. (2010), demonstrated sustained activity in regions of the DMN specifically during implicit mnemonic processing. In accordance, the deactivation during retrieval can be explained similarly by the fact that the retrieval task was operationalized as explicit in this paradigm, therefore resulting in a downregulation of the DMN network. Previous studies support the notion of a pronounced deactivation in regions relevant to the DMN during explicit memory retrieval (Daselaar et al., 2004; Marklund et al., 2007; Yang et al., 2010). Thus, the inverse E/R flips in the PMR and the left MTG represent at least in part, the different levels of cognitive effort, subjects had to exert during implicit encoding and explicit retrieval.

### 4.5 The aIC is associated with neural efficiency

In the young group, we observed a negative correlation between performance accuracy and retrieval-related activation in the aIC. This is in line with the neural efficiency hypothesis that states, that individuals with higher degrees of cognitive ability exhibit decreased levels of activation compared to individuals with lower degrees of cognitive ability (Neubauer and Fink, 2009). Even though this might be counterintuitive at first, there is mounting evidence that brain regions with high functional relevance for a specific cognitive task exhibit a negative relationship between their activation strength and the respective cognitive performance (for review, see Neubauer and Fink, 2009). The aIC, in particular, appears to be an important neurofunctional correlate in terms of neural efficiency: individuals with greater cognitive abilities demonstrated decreased levels of activation in the aIC compared to individuals with poorer cognitive abilities (Dunst et al., 2014). Thus, our finding is in line with previous evidence and supports the notion that the aIC is an interface that mediates the cognitive control network (CCN) and other related cognitive networks in response to increasing cognitive demand (Wu et al., 2019). The aIC appears to be an important neurofunctional correlate that efficiently mediates as a response to de- or increasing cognitive demand and individual cognitive ability, i.e., performance capacity.

### 4.6 Limitations concerning the investigations with aged populations

Despite our thorough methodology, naturally, our study has some limitations that should be addressed in future investigations. Until now, only a few studies investigated the effect of age on incidental learning, and even fewer studies compared incidental and intentional learning directly. The results in the present study are based on incidental learning which can account for discrepancies in prior literature, e.g., in the inverse E/R flip in the PMR. Future studies may directly compare incidental with intentional learning conditions when investigating the E/R flip and other regions of the neural memory system.

In the present study, we did not account for the influence of cardiovascular indices like blood pressure and cerebral blood flow, which have previously been shown to significantly confound the aging effects of BOLD-derived functional brain activation (Tsvetanov et al., 2021). This confound can produce putative hypo or hyperactivation. For instance, old healthy aged adults, exhibit differential BOLD signal patterns depending on whether cardiovascular influences were controlled for or not (Wu et al., 2023). However, the literature suggests that age and memory-related activation differences, e.g., in the PMR, even persist when controlling for cardiovascular indices (Braskie et al., 2010).

Lastly, we observed heterogenous neural activation, particularly within our older subject group, characterized by the co-occurence of both activation and deactivation, during our correlation analysis between aIC acitivity and performance accuracy. This could be explained by regional functional coherence in old age. Previous studies have shown that functional coherence decreases with increasing age, particularly in the insular cortex and the PMR (Klaassens et al., 2017; Eavani et al., 2018). This heterogeneity may bias some of our results, particularly those of the correlation analysis.

## 5 Conclusion and outlooks

In conclusion, the results of the current study indicate age-related neurofunctional changes in central modulatory neurocognitive processes in the associative memory system, particularly concerning the pronounced E/R flip within the aIC. Furthermore, it appears that the age-related modulatory changes in the E/R flip of the aIC are associated with significant deficits in cognitive memory performance during implicit learning and retrieval. Therefore, the impaired functional modulation of the aIC might be responsible for the maladaptive interaction of the DMN and CEN often seen in older adults during cognitive tasks, propagating the right aIC as a sensitive marker for early detection of age-related cognitive decline.

## Data availability statement

The raw data supporting the conclusions of this article will be made available by the authors upon request and without undue reservation.

## Ethics statement

The studies involving humans were approved by Ethikkomission Nordwest und Zentralschweiz (EKNZ). The studies were conducted in accordance with the local legislation and institutional requirements. The participants provided their written informed consent to participate in this study.

## Author contributions

ES: Formal analysis, Writing – review & editing, Writing – original draft, Software. MR: Formal analysis, Software, Visualization, Writing – review & editing, Writing – original draft. RK: Writing – review & editing. MC: Conceptualization, Investigation, Writing – review & editing. CR: Conceptualization, Writing – review & editing. OB: Resources, Writing – review & editing. AS: Writing – review & editing. SB: Writing – review & editing, Resources. TL: Writing – review & editing. CL: Writing – review & editing. AB: Writing – review & editing. UL: Writing – review & editing. TM: Conceptualization, Funding acquisition, Investigation, Methodology, Project administration, Resources, Supervision, Validation, Writing – original draft, Writing – review & editing.

## Footnotes

^1^http://www.bfs.admin.ch

^2^http://www.fil.ion.ucl.ac.uk/spm/
